# Advanced soft tissue visualization in conjunction with bone structures using contrast-enhanced micro-CT

**DOI:** 10.1117/1.JMI.11.6.066001

**Published:** 2024-11-22

**Authors:** Torben Hildebrand, Qianli Ma, Catherine A. Heyward, Håvard J. Haugen, Liebert P. Nogueira

**Affiliations:** a University of Oslo, Department of Biomaterials, Institute of Clinical Dentistry, Faculty of Dentistry, Oslo, Norway; b University of Oslo, Oral Research Laboratory, Institute of Clinical Dentistry, Faculty of Dentistry, Oslo, Norway

**Keywords:** contrast-enhanced micro-computed tomography, decalcification, Lugol’s iodine, soft tissues, bone

## Abstract

**Purpose:**

Micro-computed tomography (CT) analysis of soft tissues alongside bone remains challenging due to significant differences in X-ray absorption, preventing spatial inspection of bone remodeling including the cellular intricacies of mineralized tissues in developmental biology and pathology. The goal was to develop a protocol for contrast-enhanced micro-CT imaging that effectively visualizes soft tissues and cells in conjunction with bone while minimizing bone attenuation by decalcification.

**Approach:**

Murine femur samples were decalcified in ethylenediaminetetraacetic acid and treated with three different contrast agents: (i) iodine in ethanol, (ii) phosphotungstic acid in water, and (iii) Lugol’s iodine. Micro-CT scans were performed in the laboratory setup SkyScan 1172 and at the synchrotron radiation for medical physics beamline in synchrotron radiation facility Elettra. Soft and hard tissue contrast-to-noise ratio (CNR) and contrast efficiency after decalcification were measured.

**Results:**

In laboratory micro-CT, Lugol’s iodine demonstrated a threefold higher CNR in the bone marrow, representing the soft tissue portion, compared with the bone. Contrast efficiencies, measured in synchrotron micro-CT, were consistent with these findings. Higher resolutions and the specificity of Lugol’s iodine to cellular structures enabled detailed visualization of bone-forming cells in the epiphyseal plate.

**Conclusions:**

The combination of decalcification and the utilization of the contrast agent Lugol’s iodine facilitated an enhanced soft tissue visualization in conjunction with bone.

## 1 Introduction

Micro-computed tomography (micro-CT) analysis is a highly valuable tool for investigating bone and other mineralized tissues.^1^^–^^3^ The indispensability of the methodology is explained by its ability to provide high-resolution, three-dimensional, non-invasive, relatively low-cost images.^4^^,^^5^ The technique has revolutionized the study of bone microarchitecture, allowing detailed visualization of the trabecular and cortical bone structures. However, micro-CT imaging of non-mineralized tissues, such as the muscles, tendons, and cartilage, presents significant challenges. These soft tissues’ intrinsic X-ray absorption constrains conventional micro-CT methods to visualize hard tissues only.^6^

Contrast enhancement agents have been investigated to improve the visibility of soft tissues in micro-CT imaging. Those contrast agents bind to the biological material and increase the contrast due to the high atomic number (Z) and its proportional X-ray attenuation.^7^^,^^8^ Nowadays, iodine-based solutions and phosphotungstic acid (PTA) are widely utilized for contrast-enhanced micro-CT and were first described by Metscher.^9^^,^^10^ This advancement has opened new possibilities for detailed examination of the soft tissue structures, which are crucial for understanding the complexity of structural interactions of the bone in its musculoskeletal system, including soft tissues and cells.

Bone tissue as a dynamic system is characterized by the interplay of various tissues and cells that ensure its functionality. The ligaments, tendons, and cartilage work together to facilitate the movement and stability of the musculoskeletal system,^11^^–^^13^ whereas cellular components such as osteoblasts, osteoclasts, and osteocytes are actively involved in bone remodeling and maintenance.^14^^–^^16^ Traditional methods for studying these components include classical histology utilizing hematoxylin and eosin (H&E), Masson–Goldner trichrome, modified Masson–Goldner trichrome, Movat’s pentachrome, and alcian blue staining to identify bone healing status and the present cell types.^17^ Although highly specific, these methods involve labor-intensive and time-consuming preparation of the sample.^18^^–^^20^ Further, approaches to resolve three-dimensional analysis require very complex serial sectioning of the tissue and reconstruction of these.^18^ Micro-CT, as a tomographic imaging technique, provides high-resolution images to resolve intricate structures spatially.

Despite the potential of contrast-enhanced micro-CT (CE micro-CT), several challenges remain. One significant issue is the high attenuation of X-rays by mineralized bone, which can overshadow the enhanced contrast of adjacent soft tissues. High contrast enhancement along calcified bone also requires higher tube powers, which causes focal spot enlargement.^21^ Increased focal spot sizes can result in a loss of resolution and detail for the soft tissue structures, undermining the overall effectiveness of the imaging technique. A decalcification process can be employed prior to contrast enhancement. Decalcification involves removing the mineral content from the bone, thereby reducing its X-ray attenuation and allowing better visualization of soft tissues and cellular structures due to lower tube powers and reduced focal spot sizes.

The primary objective of this study is to develop and optimize a protocol for CE micro-CT imaging that enhances the visualization of soft tissues and cells in conjunction with the bone. This involves assessing the effectiveness of different contrast enhancement agents—iodine in ethanol (I2E), PTA, and Lugol’s iodine—in improving the contrast of decalcified bone and associated soft tissues. The study utilizes femur samples and tibiofemoral joints from murine models to evaluate the contrast enhancement efficiency of these agents.

This study aims to significantly enhance the capability of micro-CT imaging for detailed spatial investigations into the cellular and soft tissue composition of bone. By combining decalcification with contrast enhancement, the protocol will likely allow for improved visualization of soft tissue structures within the bone. This has important implications for advancing our understanding of bone biology, pathology, and the complex interactions among different tissue types within the skeletal system or prospectively biomaterials.

## 2 Methodology

### 2.1 Sample Preparation

**Fig. 1:**
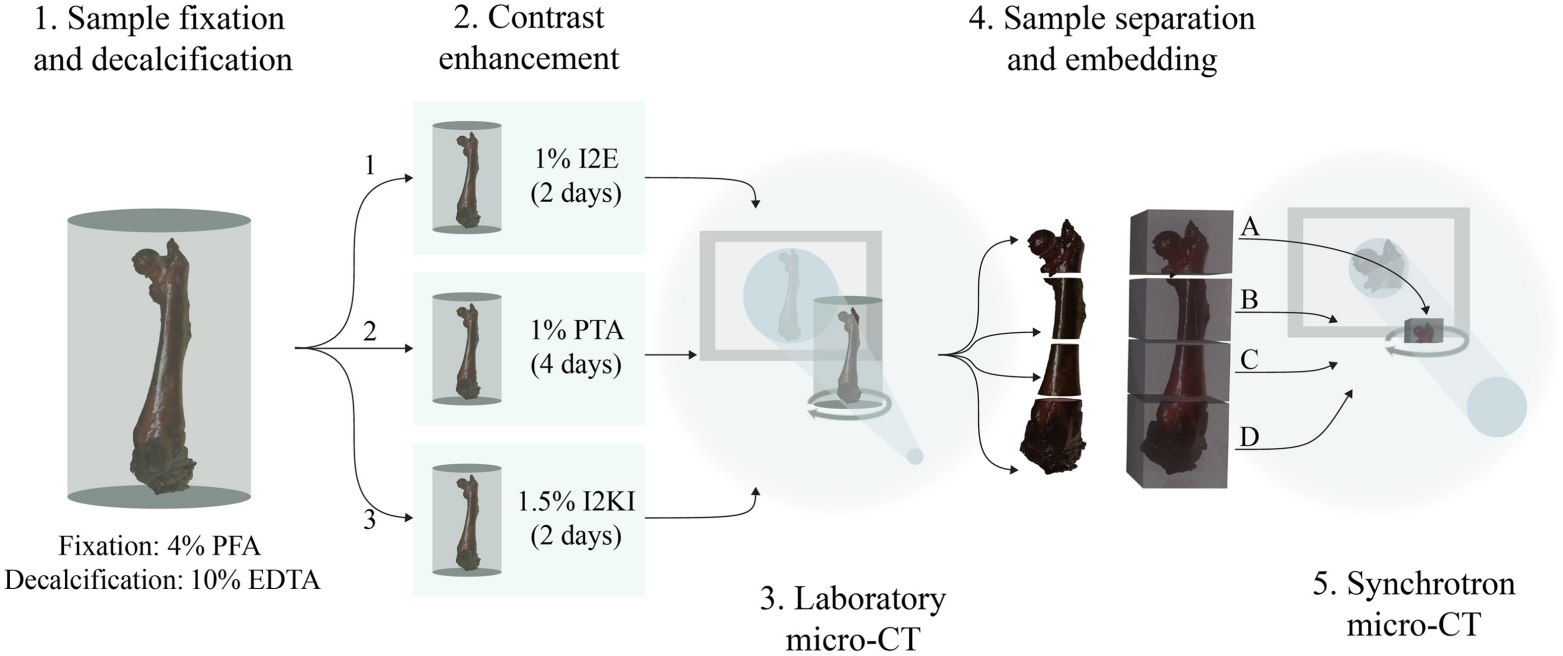
Preparation of femur samples. After fixation in 4% PFA, samples were decalcified in 10% EDTA and then contrast-enhanced in indicated contrast agents and concentrations for 2 or 4 days (1–3). Then, the sample was cut into four samples; two represent the joints (A and D), and the other two are the cortical bone (B and C). Finally, samples were embedded in paraffin after dehydration and clearing.

Eight femur samples from four 28-day-old B6 mice (wild type) were harvested. Subsequently, the femur samples were immediately fixed in 4% paraformaldehyde (PFA) and immersed in 10% ethylenediaminetetraacetic acid (EDTA) (pH 7.25) for decalcification for 14 days. One femur remained calcified in phosphate buffered saline as a control (n=1), and another control sample was decalcified but remained without contrast enhancement (n=1). The other femur samples were divided into three groups: (i) 1% iodine in ethanol (n=2), (ii) 1% PTA in water (n=2), and (iii) 1.5% Lugol’s iodine [0.5% iodine ($I_{2}$), 1% potassium iodide] (n=2). Group i was adapted to 70% ethanol in 10% steps, each step lasting for 15 min, whereas groups ii and iii were transferred to distilled water prior to contrast enhancement. Immersion times amounted to 2 days for iodine-based and 4 days for PTA-based contrast enhancement at room temperature. After contrast enhancement, samples from group i were rinsed and scanned in 70% ethanol and groups ii and iii in distilled water. Imaging in a laboratory micro-CT setup was performed as described in Sec. 2.2, and the scanned samples and the control samples (calcified and decalcified without contrast enhancement) were divided into four parts, as illustrated in Fig. 1. All samples (A to D) were then adapted to 100% ethanol in 10% steps for 15 min and embedded in type 9 paraffin wax (Epredia, Kalamazoo, Michigan, United States) after xylene clearing. Then, synchrotron micro-CT scans were performed as described in Sec. 2.3.

### 2.2 Laboratory Micro-CT

Samples were scanned between steps 2 and 4, as illustrated in Fig. 1 utilizing Skyscan 1172 (Bruker Belgium, Kontich, Belgium) at 51 kV and 169  μA with an exposure time of 750 ms, and a rotation step of 0.97 deg over 360 deg, leading to a voxel size of 13.46  μm. The scan duration was ∼20  min. Reconstructions were performed with the system-provided software NRecon (version 1.7.4.6) with ring artifact correction 10 and beam hardening correction of 40%.

### 2.3 Synchrotron Micro-CT

After accomplishing step 4 in Fig. 1, samples were scanned at the synchrotron radiation for medical physics (SYRMEP) beamline in the synchrotron laboratory Elettra (Trieste, Italy) using a sample-to-detector distance of 100 mm and a 0.5-mm silicon filter. The projections were obtained with 16.7 keV and 308 mA and an exposure time of 50 ms over 180 deg and 2400 projections. The pixel size was set to 0.9  μm. Acquisition times were ∼3  min for each scan. The joints (samples A and D from each group) were scanned over 360 deg with 3600 projections, leading to increased acquisition times of ∼4.5  min. Reconstructions were performed using STP (version 1.6.2), a custom reconstruction program of SYRMEP.

### 2.4 Contrast Assessment

Scans from laboratory micro-CT were analyzed regarding its bone marrow (BM) to bone (B) contrast after contrast enhancement. Gray values of BM and B were measured in each of the 12 small areas of interest in the corresponding tissue with areas of ∼20,000 and $5000\;{\mu m}^{2}$ and gray values of the background (Bkg) in 12 areas of each $0.3\;{\mathrm{mm}}^{2}$. The contrast-to-noise ratio (CNR), as defined by Bushberg and Boone,^22^ was calculated for bone tissue, bone marrow, and the difference between the bone marrow and the bone (BM − B). This analysis aimed to assess the effectiveness of each contrast agent for visualizing B and BM, as well as the contrast between these two structures, as follows:

$${\mathrm{CNR}}_{B}=\frac{{\overset{¯}{\mathrm{GV}}}_{B}-{\overset{¯}{\mathrm{GV}}}_{\mathrm{Bkg}}}{{\mathrm{SD}}_{\mathrm{Bkg}}},$$ (1)

$${\mathrm{CNR}}_{\mathrm{BM}}=\frac{{\overset{¯}{\mathrm{GV}}}_{\mathrm{BM}}-{\overset{¯}{\mathrm{GV}}}_{\mathrm{Bkg}}}{{\mathrm{SD}}_{\mathrm{Bkg}}},$$ (2)

$${\mathrm{CNR}}_{\mathrm{BM}-B}=\frac{{\overset{¯}{\mathrm{GV}}}_{\mathrm{BM}}-{\overset{¯}{\mathrm{GV}}}_{B}}{{\mathrm{SD}}_{\mathrm{Bkg}}},$$ (3)

where $\overset{¯}{\mathrm{GV}}$ is the average gray value of the indicated tissue bone, bone marrow, or the surrounding medium ethanol or water background, and the divisor indicates the standard deviation (SD) of the gray values in the background.

Scans from synchrotron micro-CT were analyzed regarding its bone marrow and bone contrast enhancement as follows: gray values for each contrast agent were measured in the bone marrow and bone tissue for each group in 12 areas of the same sizes as for laboratory micro-CT analysis from eight different scans. Gray values of the according tissue after decalcification and prior contrast enhancement were utilized to calculate the relative increase:

$$E_{\mathrm{CE}}=\frac{{\overset{¯}{\mathrm{GV}}}_{\mathrm{CE}}-{\overset{¯}{\mathrm{GV}}}_{\text{decalcified}}}{{\overset{¯}{\mathrm{GV}}}_{\text{decalcified}}}\cdot 100\%,$$ (4)

where $E_{\mathrm{CE}}$ describes the efficiency of contrast enhancement for tissue after decalcification. ${\overset{¯}{\mathrm{GV}}}_{\mathrm{CE}}$ indicates the average gray value of the tissue with contrast enhancement, and ${\overset{¯}{\mathrm{GV}}}_{\text{decalcified}}$ indicates before contrast enhancement and after decalcification. Ratios of contrast enhancement efficiencies of bone marrow to bone were then calculated. For values >1, the bone marrow was enhanced, and for values <1, the bone was enhanced. Values close to 1 indicate a balanced gray value distribution among the tissues.

Gray values were measured, and illustrations were generated in Dragonfly 3D World (Comet Technologies Canada Inc., Montréal, Canada, version 2024.1, Build 1601).

### 2.5 Histology

Histological sections were obtained from the isolated samples after synchrotron micro-CT imaging. Samples were placed into base molds, oriented, and embedded into paraffin blocks. Sections from the paraffin block of 7  μm thickness were obtained with Leica RM2255 rotary microtome (Leica Biosystems Nussloch GmbH, Nussloch, Germany) and placed on Superfrost positively charged glass slides. Subsequently, the slides were baked, deparaffinized, rehydrated, and stained with H&E.

## 3 Results

### 3.1 Laboratory Micro-CT

**Fig. 2:**
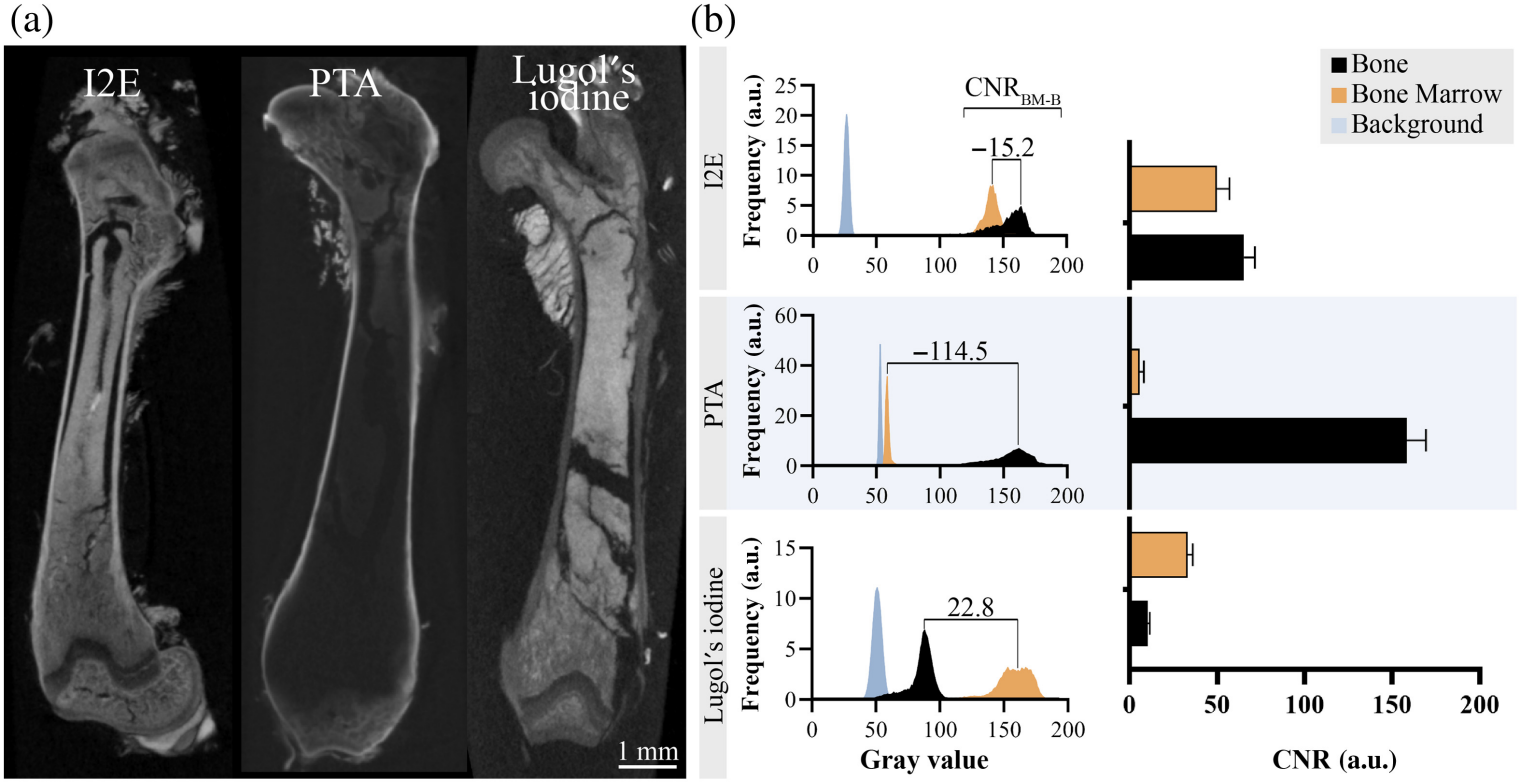
Micro-CT scans of contrast-enhanced femur samples after decalcification (a). (b) Corresponding gray value distribution and CNR measurements for I2E, PTA, and Lugol’s iodine. Values are the mean ± SD from 12 area measurements.

Scans from mouse femur after decalcification and contrast enhancement utilizing I2E, PTA, and Lugol’s iodine are illustrated in Fig. 2. The bone and bone marrow after applying I2E indicate similar gray values. Corresponding histograms showed higher values within the bone. PTA as a contrast agent increased the gray values of the bone strongly. However, it left a diffusion gradient and did not distribute evenly. On the other hand, the employment of Lugol’s iodine increased the gray values of the bone marrow to a higher extent than the decalcified bone.

Contrast-to-noise ratios calculated by Eqs. (1)–(3) demonstrated the highest values for the bone contrast-enhanced with PTA and the lowest for the bone marrow utilizing PTA. As a contrast agent in ethanol, iodine achieved higher CNR for both bone and bone marrow than Lugol’s iodine. However, ${\mathrm{CNR}}_{B}$ was relatively low utilizing Lugol’s iodine and not significantly higher than ${\mathrm{CNR}}_{\mathrm{BM}}$ with PTA contrast enhancement.

${\mathrm{CNR}}_{\mathrm{BM}-B}$ was negative for I2E (−15.2) and PTA (−114.5) contrast enhancement, whereas Lugol’s iodine expressed a higher value (22.8).

### 3.2 Synchrotron Micro-CT

**Fig. 3:**
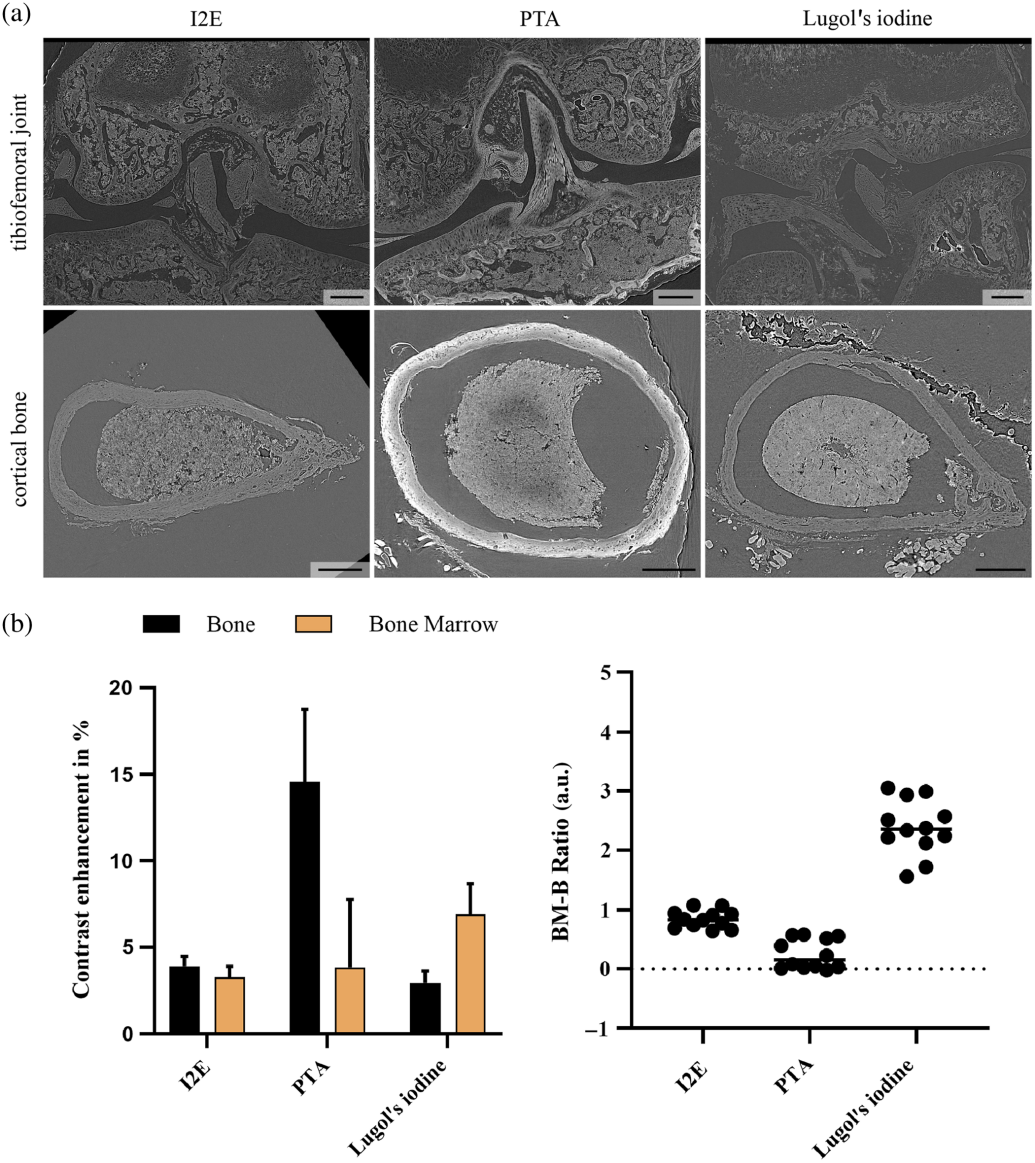
(a) Comparison of contrast enhancement agents after decalcification in the murine tibiofemoral joint and cortical bone. I2E showed a similar contrast enhancement in the bone marrow and bone, whereas PTA increased predominately in the decalcified bone. Lugol’s iodine enhanced the soft tissue, including the bone marrow, to a higher extent than bone. Measurements indicate this observation (b), leading to a bone marrow-to-bone ratio >1 for Lugol’s iodine. Values are the mean ± SD from 12 area measurements from eight scans from two femurs in each group.

The contrast for the bone was dramatically increased using PTA, and the bone marrow was enhanced to a similar degree as iodine-enhanced samples. It must be noted that the standard deviations of the contrast enhancement are higher for PTA samples than for the iodine-enhanced ones. Figure 3 demonstrates the effect of each contrast agent for a cross-section in the tibiofemoral joint and cortical bone. Lugol’s iodine was the only contrast agent that showed stronger contrast enhancement in the bone marrow than in the bone. The ratio of the contrast efficiencies from the bone marrow (BM) to the bone (B) using Luogl’s iodine is ∼2.5, whereas the ratio falls below 1 for the other agents. This indicates that only Lugol’s iodine can differentially enhance the contrast of the bone marrow above that of the bone.

**Fig. 4:**
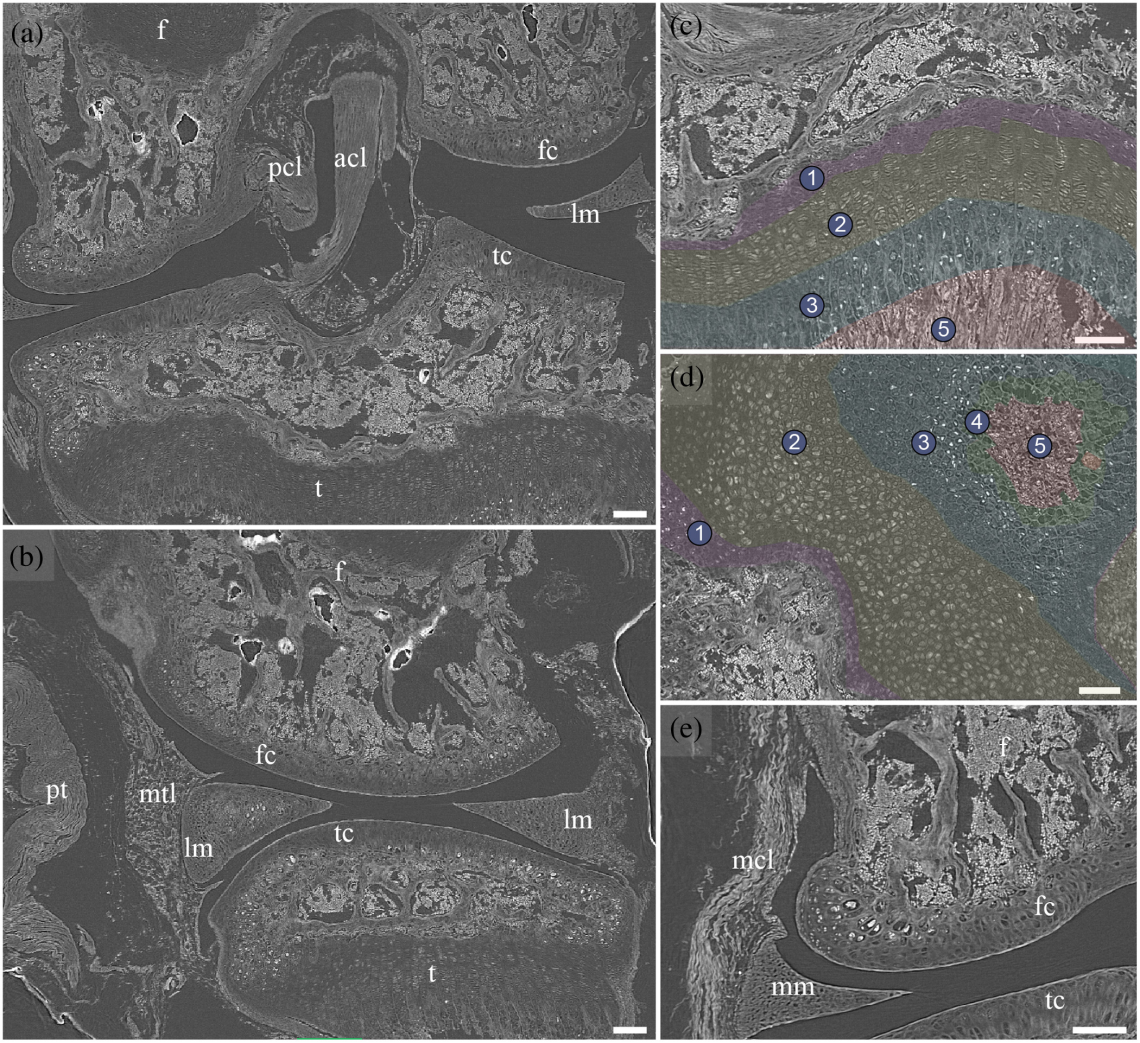
Murine tibiofemoral joint contrast-enhanced with 1.5% Lugol’s iodine after decalcification in different planes. (a) Coronal plane. (b) Sagittal plane. Magnification of the proximal tibia growth plate in the (c) coronal plane and (d) transverse plane. The zone of resting cartilage (1), proliferation (2), hypertrophy (3), calcification (4), and ossification (5) are visualized. (e) Magnification of the femoral articular cartilage in the coronal plane. acl, anterior cruciate ligament; f, femur; fc, femoral cartilage; lm, lateral meniscus; mcl, medial collateral ligament; mm, medial meniscus; mtl, meniscotibial ligament; pcl, posterior cruciate ligament; pt, patellar tendon; t, tibia; tb, tibial cartilage. Scale bars: 100  μm.

Figure 4 demonstrates multiple planes of the tibiofemoral joint, contrast-enhanced by Lugol’s iodine. The bone marrow along the epiphyseal plate is highlighted in the coronal and sagittal planes [Figs. 4(a) and 4(b)]. However, the ligaments, other connective tissues, meniscus, and trabecular bone are identifiable. Magnifications of the growth plate in the coronal and transversal planes highlight the presence of characteristic arrangement of the chondrocytes. Although the zone of the resting cartilage is only rudimentarily observable (1), the zone of proliferation, with aligned columns of disc-shaped cells, is clearly visualized (2). The zone of the hypertrophic cartilage is visible by stronger enhanced spherical chondrocytes (3). The chondrocytes increase further in contrast to the newly forming bone matrix (5). In contrast, a gap in the presence of chondrocytes (4) can be found in the transversal plane between the hypertrophic cartilage (3) and the newly forming bone matrix (5). The transversal plane of the epiphyseal plate [Fig. 4(d)] visualizes the arrangement of the chondrocytes as a round disk from the top of the disc-shaped cells in the zone of proliferation. The matrix embedding of the chondrocytes is becoming increasingly visible from zones 2 to 4. The section from the secondary ossification center is displayed in Fig. 4(e).

### 3.3 Histology

**Fig. 5:**
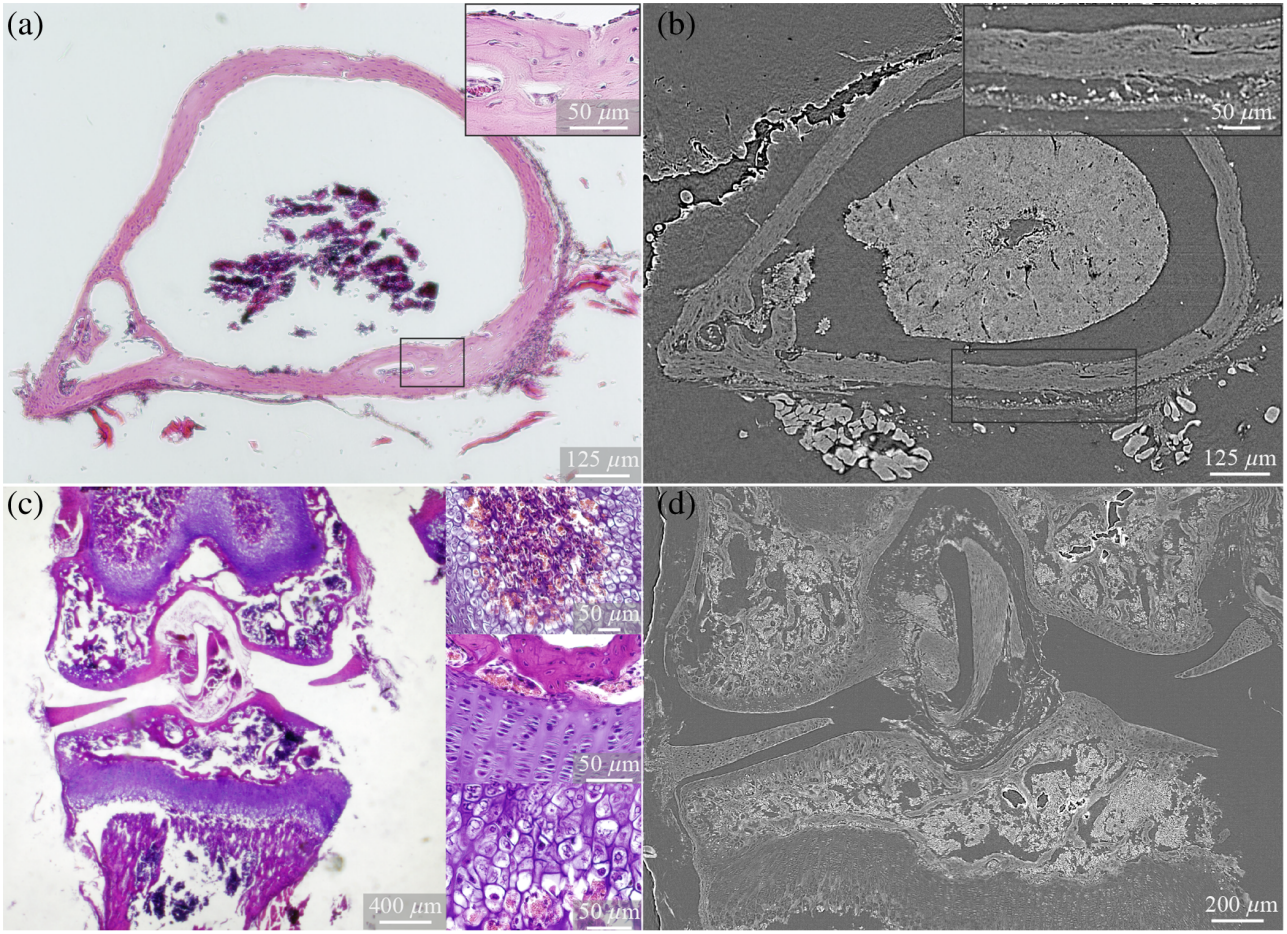
Comparison of the cortical bone with H&E staining (a) and with contrast-enhanced micro-CT employing Lugol’s iodine (b) and of the tibiofemoral joint accordingly (c), (d). Sectioning of the paraffin-embedded bone caused distortions of the tissues, especially for the bone marrow, compared with micro-CT imaging. Osteocytes in the embedded bone matrix were visible in the H&E sections but could not be seen in micro-CT sections.

Histological sections from previously scanned samples confirmed the tissue structures observed in the micro-CT imaging and exhibited similar morphological characteristics. However, the histological sectioning method caused tissue distortion, particularly affecting the shape of the cortical bone and the integrity of the soft tissues, including the enclosed bone marrow. Although osteocytes were not visible in the micro-CT images, H&E staining confirmed their presence within the bone matrix [Figs. 5(a) and 5(b), magnification].

## 4 Discussion and Conclusion

This study focused on evaluating the three contrast agents I2E, PTA, and Lugol’s iodine after the decalcification in murine femur samples. Laboratory micro-CT, utilizing a voxel size of 13.46  μm and imaging the entire femur, as well as synchrotron micro-CT with voxel sizes of 0.9  μm in isolated samples with a height of ∼1.7  mm and a diameter of 2.6 mm, resulting in volumes of $∼9\;{\mu m}^{3}$, was performed. Although the laboratory micro-CT imaging illustrated the global contrast agent distribution, synchrotron micro-CT aimed to unveil the ultrastructural features of the bone samples.

Micro-CT imaging of the entire femur demonstrated the distribution of the gray values after contrast enhancement within the decalcified samples. Although PTA and I2E resulted in a higher CNR in the bone, Lugol’s iodine resulted in a higher CNR in the bone marrow; however, the CNR was lower than the I2E- and PTA-based contrast enhancement. This may have two reasons—first, the concentration of iodine in Lugol’s iodine amounted effectively to 0.5%, whereas in I2E, it amounted to 1% iodine in the contrasting solution. This may have resulted in varying levels of tissue saturation by the contrast agent. The concentration-dependent potential for contrast enhancement was reported for iohexol, an iodinated contrast medium. With increasing concentration, the enhancement increased, particularly in lower tube potentials of 40 to 60 kVp.^23^ The concentration of PTA was also 1% in the contrasting solution; however, it has shown higher induced contrast increases than iodine-based contrast agents in various samples such as *Drosophila melanogaster* or the oval squid brain.^24^^,^^25^ Metscher^10^ observed higher gray values for 0.3% PTA than for 1% I2E or 10% Lugol’s iodine (1% iodine and 2% potassium iodide) in the hindlimb buds of stage 24 chick embryos. Conversely, the bone marrow remained in uneven gray values comparable with the background. It thus indicated a ${\mathrm{CNR}}_{\mathrm{BM}}$ of 5.7±2.5 which could point to an insufficient contrast duration to diffuse throughout the decalcified bone, even though an increased penetration rate of the contrast agent in decalcified tissue was anticipated. Second, the medium surrounding the sample during the image acquisition was ethanol in the case of I2E. At the same time, PTA and Lugol’s iodine were rescanned in water, which attenuates X-rays more than ethanol.^26^^,^^27^ This is reflected in the shift of the backgrounds’ gray values in Fig. 2 toward higher values for PTA and Lugol’s iodine, which leads to a higher background noise (${\overset{¯}{\mathrm{GV}}}_{\mathrm{Bkg}}/{\mathrm{SD}}_{\mathrm{Bkg}}$) and, subsequently, to a lower CNR.

Lugol’s iodine enhanced the soft tissue, represented by the bone marrow, to a greater extent than the decalcified bone which indicates that the contrast agent is a promising agent for soft tissue enhancement in conjunction with bone. Similar characteristics were described previously by Li et al.:^28^ although I2E enhanced calcified bone significantly more than Lugol’s iodine, contrast enhancement with Lugol’s iodine increased the contrast of most of the observed soft tissues to a similar extent to I2E. Albeit CNRs for Lugol’s iodine were comparatively low, gray values of the bone marrow were in a resembling range than the bone and bone marrow utilizing I2E. In contrast, gray values for the bone remained lower, which increases the contrast between these tissues, expressed by the ${\mathrm{CNR}}_{\mathrm{BM}-B}$ of 22.8. Only the ${\mathrm{CNR}}_{\mathrm{BM}-B}$ value for PTA was higher (114.5). However, the CNR for bone marrow was close to 0, which does not allow for distinguishing.

Results from the synchrotron micro-CT setup agreed with those from the laboratory micro-CT, confirming that, of the contrast agents tested, only Lugol’s iodine could enhance the soft tissue portion to a greater extent than the decalcified bone. Here, the contrast enhancement was measured in percentage based on the corresponding tissue after decalcification and without contrast enhancement, which was facilitated by the synchrotron micro-CT using the phase contrast for structure identification, which can enhance the soft tissue contrast of non-contrast-enhanced samples.^29^ The ratio of contrast enhancement of the bone marrow to the bone was ∼2.5 using Lugol’s iodine, whereas I2E and PTA were below 1, highlighting Lugol’s iodine as a promising agent for soft tissue enhancement alongside bone. It must be noted that samples treated with Lugol’s iodine were quickly dehydrated using ethanol for paraffin embedding. This could have influenced the mentioned ratio negatively by changing the predominantly triiodide ions in Lugol’s iodine into iodide ions and reversing the binding characteristics from Lugol’s iodine toward I2E. Further, contrast enhancement for bone utilizing PTA remained comparatively high, whereas contrast enhancement for I2E was found to be <5%, less than anticipated from results shown for laboratory micro-CT. Diffusion of the contrast agent due to dehydration to 100% ethanol and paraffin embedding might explain this observation, which occurs to a lesser extent with PTA contrast enhancement. Contrast durability has been shown to be higher for PTA than for iodine-based contrast enhancement in hydrogel embedding.^24^ This may occur to an increased degree in ethanol, explaining decreased contrast efficiencies in iodine-based contrast enhancement.

Besides the presented effects of the contrast enhancement after decalcification in EDTA, the similarities of the obtained images to classical H&E staining are notable. Although conventional histological approaches can be challenging in preventing tissue distortion that occurs due to the compressive strains induced by mechanical sectioning and shrinkage due to fixation, dehydration, and embedding,^30^ micro-CT imaging prevents these distortions by virtual sectioning. However, influences from fixation cannot be avoided, scanning in paraffin further increases the risk of underestimating the samples’ dimensions, whereas after rehydration in microscopic slides, the samples’ dimensions can be restored until approximately the shrinkage from the prior fixation.^31^ Micro-CT scans of paraffin-embedded human and murine soft tissues have decreased volumes by 19.2% to 61.5%.^32^ Lastly, Lugol’s iodine as a contrast agent has shown additional tissue shrinkage depending on its concentration, tissue type, incubation time, and fixative for soft tissues from BL6 mice.^33^ Vickerton et al.^33^ found shrinkage rates for 2% Lugol’s iodine solution to be ∼10% for the cerebellum, skeletal, and cardiac muscle of BL6 mice after 1 day of incubation. In addition, Dawood et al.^34^ have demonstrated that decreasing pH inducing significantly soft tissue shrinkage in mouse liver. For this reason, an increased shrinkage rate for scanning in paraffin embedding in synchrotron micro-CT can be expected to be significant, but it is not quantified. A further limitation results from the osteocytes embedded in the bone matrix, which were not visualized with the presented approach, whereas they were present in light microscopy after H&E staining. For an explanation, the contrast agents’ characteristics with the specific cell type must be investigated further, assuming that the contrast agent could diffuse throughout the decalcified bone and reach the cells. On the other hand, chondrocytes that underwent cell death in zone 4 of Fig. 4 are not further visible but can be observed in classical histology. Up to this point, chondrocytes increased in contrast and hence showed uptake of the contrast agent (zone 3). In this zone of hypertrophy, chondrocytes in chick bone were found to steadily increase glycogen content,^35^^,^^36^ which could explain the observed increase in contrast because it has been previously described that Lugol’s iodine may be bound to glycogen.^28^^,^^37^ In the degenerating chondrocytes, the glycogen content was reduced;^32^ this may explain why the chondrocytes, after cell death, were no longer visible in micro-CT images. This observation might lead to promising insights into the process of endochondral ossification utilizing contrast-enhanced micro-CT with Lugol’s iodine and prior decalcification.

The limitation of the findings is presented in the assessment of PTA, as it can be observed that it is not homogenously distributed within the sample. Its relatively larger molecule compared with iodine-based contrast agents such as I2E or Lugol’s iodine can explain this.^38^ Even though PTA binds to decalcified bone effectively, it could be disputable that the CNR for the bone marrow is represented as too low. The higher molecular weight of PTA and the dense collagenous matrix of the bone could have decreased the agent’s diffusion through the sample. Longer immersion durations in the contrast agent should be tested. However, its strong binding affinity to the decalcified bone matrix reversed the effect of the decalcification regarding X-ray attenuation, reducing the potential for soft tissue enhancement relative to bone.

Further limitations remain in the resolutions achieved in micro-CT systems for quantitative cell analysis, e.g., cell counting, for more advanced assessment of possible interventions. Future investigations could utilize systems with higher resolution capabilities with the demonstrated protocol in mineralized tissues.

An additional limitation is the utilization of general contrast enhancement agents, usually selected based on contrast agents known from electron microscopy,^8^ whose exact contrasting characteristics for micro-CT are investigated after employment. Tailored contrast agents specifically binding to target molecules or cell components, for instance, binding to the nucleus, could conclude that binding to the nucleus could result in more sophisticated results, such as immunohistochemical staining, but three-dimensional. First target specific protocols were reported by Müller et al.^39^ and Metscher;^40^ however, a combination of these protocols or different targets have to be elaborated, which demonstrates enormous efforts and indicates a promising research direction for the future. The nucleus-specific contrasting protocol implies the potential to enhance the contrast of osteocytes that could not be visualized with Lugol’s iodine and might improve cell visibility along bone after decalcification.

A general drawback of the described approach is that information about bone calcification is lost during decalcification, limiting this method primarily to enhanced visualization of soft tissues. However, it is possible to integrate this approach with conventional micro-CT, merging hard and soft tissue data, as previously demonstrated in studies of mouse mandible soft and hard tissue analysis.^41^

The non-invasive micro-CT enables, compared with traditional methods, the visualization of different planes simultaneously and allows the subsequent performance of classical methods. Micro-CT scans, contrast-enhanced with the described properties of predominantly binding to the cellular components, enable artificial colorings, such as mimicking classical H&E staining shown in Video 1.

This technique provides information about spatial cellular arrangements in bone and potentially other mineralized tissues, offering a pre-histological assessment and optimizing the performance of classical histology. However, it is important to note that the decalcification duration must be significantly extended when applying this protocol to human bones or larger samples. In addition, if geometrical magnification is employed, the resolution of larger samples may decrease, potentially leading to a loss of cellular detail.

In conclusion, decalcification combined with contrast enhancement in micro-CT imaging is a promising methodology for enhancing soft tissue visualization in conjunction with bone structures. Lugol’s iodine has shown advantageous binding properties to soft tissue components, exceeding the contrast of bone structures and enabling an improved discrimination.

## 5 Appendix: Video File

Video 1. Multi-orientation virtual histology mimicking H&E staining (MP4, 11.7 MB [URL: https://doi.org/10.1117/1.JMI.11.6.066001.s1]). After orientating the sample virtually, a H&E mimicking look-up table was applied. Several planes can be illustrated simultaneously, e.g., a transversal section of the femur in the height of the growth plate (green), sagittal (red), and coronal section (blue) of the tibiofemoral joint.

## Supplementary Material



## Data Availability

All data in support of the findings of this paper are available within the article.
